# Antibacterial Nanostructured Ti Coatings by Magnetron Sputtering: From Laboratory Scales to Industrial Reactors

**DOI:** 10.3390/nano9091217

**Published:** 2019-08-28

**Authors:** Rafael Alvarez, Sandra Muñoz-Piña, María U. González, Isabel Izquierdo-Barba, Iván Fernández-Martínez, Víctor Rico, Daniel Arcos, Aurelio García-Valenzuela, Alberto Palmero, María Vallet-Regi, Agustín R. González-Elipe, José M. García-Martín

**Affiliations:** 1 Instituto de Ciencia de Materiales de Sevilla (CSIC-US), Américo Vespucio 49, 41092 Seville, Spain; 2 Departamento de Física Aplicada I, Escuela Politécnica Superior, Universidad de Sevilla, c/Virgen de África 7, 41011 Seville, Spain; 3 Nano4Energy SLNE, C/Jose Gutierrez Abascal 2, 28006 Madrid, Spain; 4 Instituto de Micro y Nanotecnología, IMN-CNM, CSIC (CEI UAM + CSIC), Isaac Newton 8, 28760 Tres Cantos, Madrid, Spain; 5 Dpto de Química en Ciencias Farmacéuticas, Facultad de Farmacia, Universidad Complutense de Madrid, Instituto de Investigación Sanitaria Hospital 12 de Octubre i+12, Plaza Ramón y Cajal s/n, 28040 Madrid, Spain; 6 CIBER de Bioingeniería Biomateriales y Nanomedicina (CIBER-BBN), 28029 Madrid, Spain

**Keywords:** magnetron sputtering, oblique angle deposition, nanostructured titanium thin films, antibacterial coatings, osteoblast proliferation, industrial scale

## Abstract

Based on an already tested laboratory procedure, a new magnetron sputtering methodology to simultaneously coat two-sides of large area implants (up to ~15 cm^2^) with Ti nanocolumns in industrial reactors has been developed. By analyzing the required growth conditions in a laboratory setup, a new geometry and methodology have been proposed and tested in a semi-industrial scale reactor. A bone plate (DePuy Synthes) and a pseudo-rectangular bone plate extracted from a patient were coated following the new methodology, obtaining that their osteoblast proliferation efficiency and antibacterial functionality were equivalent to the coatings grown in the laboratory reactor on small areas. In particular, two kinds of experiments were performed: Analysis of bacterial adhesion and biofilm formation, and osteoblasts–bacteria competitive in vitro growth scenarios. In all these cases, the coatings show an opposite behavior toward osteoblast and bacterial proliferation, demonstrating that the proposed methodology represents a valid approach for industrial production and practical application of nanostructured titanium coatings.

## 1. Introduction

Addressing the problem of infection from the very first stage, i.e., inhibiting the formation of the bacterial biofilm, is a crucial step to prevent bone implant rejection. Recent studies indicate that nanostructured surfaces can be a less aggressive alternative to antibiotics to avoid infections [1,2], with the additional advantage of improving the behavior of osteoblasts, the cells that regenerate bone [3,4]. In this regard, the fabrication of nanostructured surfaces that may simultaneously favor the growth of osteoblasts and hinder bacterial proliferation represents a milestone in this research field with important implications, not only regarding the quality of life of patients but also by promoting a new generation of orthopedic implants. In the last few years, various alternatives have been proposed to induce such selective behavior, either by using nanostructures that incorporate drugs or bactericidal elements such as silver [5,6,7], or by surface processing with a strong corrugation at the nanoscale [8,9]. In our earlier work in 2015, we manufactured nanostructured coatings made of titanium (Ti) nanocolumns by oblique angle deposition (OAD) with magnetron sputtering onto the surface of Ti-6Al-4V discs, one of the alloys most commonly used in orthopedic implants. In vitro experiments showed that these nanocolumnar Ti coatings exhibited an efficient antibacterial behavior against *Staphylococcus aureus* (the bacterial adhesion decreased, and biofilm formation was prevented) without altering their biocompatibility (the osteoblasts proliferated and retained their mitochondrial activity) [10]. Moreover, in a more recent work, we have shown that these coatings also render similar antibacterial functionality against gram-negative bacteria (*Escherichia coli*) and, what is more important in order to have a direct impact in the field of medical implants, that these coatings could be prepared on small areas (~1 cm^2^) either in a laboratory setup or in a semi-industrial scale equipment [11].

In this paper, we analyze the practical use of these coatings and their fabrication on larger scales, an aspect that is mandatory for the development of actual applications [12]. In general, regarding the minimization of costs and other economic and throughput issues, turning laboratory-size devices into operational market-ready products is a crucial engineering challenge that requires scaling up laboratory procedures to large area and mass production [13]. This issue is quite evident when using the magnetron sputtering (MS) method: By this technique, a plasma is made to interact with a solid target in a vacuum reactor, producing the sputtering of atomic species from a well-defined race-track region, and their deposition on a substrate located a few centimeters away [14]. In a classical MS configuration, the substrate is placed parallel to the target, producing the growth of highly compact and dense coatings, in a process that has been easily scaled up to mass-production methods by simply building larger versions of laboratory reactors [15]. Following this methodology, the magnetron sputtering technique has demonstrated being of great utility for the production of market ready devices in microelectronics [16], optical coatings [17], or sensors [18], among other devices and products [19,20,21,22,23]. Unlike the classical configuration, the OAD geometry promotes the arrival of sputtered atoms at the substrate along an oblique direction, inducing surface shadowing mechanisms and the formation of nanocolumnar arrays, which has been usually achieved by rotating the substrate with respect to the target in laboratory-scale procedures. However, due to the strongly non-linear nature of these atomistic processes, scaling up the OAD methodology from laboratory to mass-production scales is not straightforward, requiring the development of new approaches [24,25] and reactor designs [26], issues that have scarcely been addressed in the literature [27,28].

In this line, herein we develop a new engineering approach to coat with Ti nanocolumns two sides of bone plates with areas up to ~15 cm^2^ that are commonly used to immobilize bone segments, and would be adequate for the development of this and other biomedical applications. To set up this new methodology we proceeded in the following way: We first analyzed the fundamental conditions leading to the formation of the nanocolumnar structures in a laboratory reactor, in particular, the energy and angular distribution of sputtered particles ejected from the magnetron target; then, based on these results, we proposed a new geometry to operate at oblique angles in semi-industrial reactors that reproduces these energy and momentum distributions at much larger scales. To prove the feasibility of the proposed design, we homogeneously and simultaneously coated the two sides of relatively large substrates and analyzed whether the antibacterial functionalities were the same as those obtained on surfaces manufactured in a laboratory MS reactor. In particular, two kinds of experiments were performed: Bacterial adhesion and biofilm formation, and osteoblasts–bacteria competitive in vitro assays, the latter also named the “Race for the Surface” competition [29].

## 2. Experimental Setup

**Figure 1:**
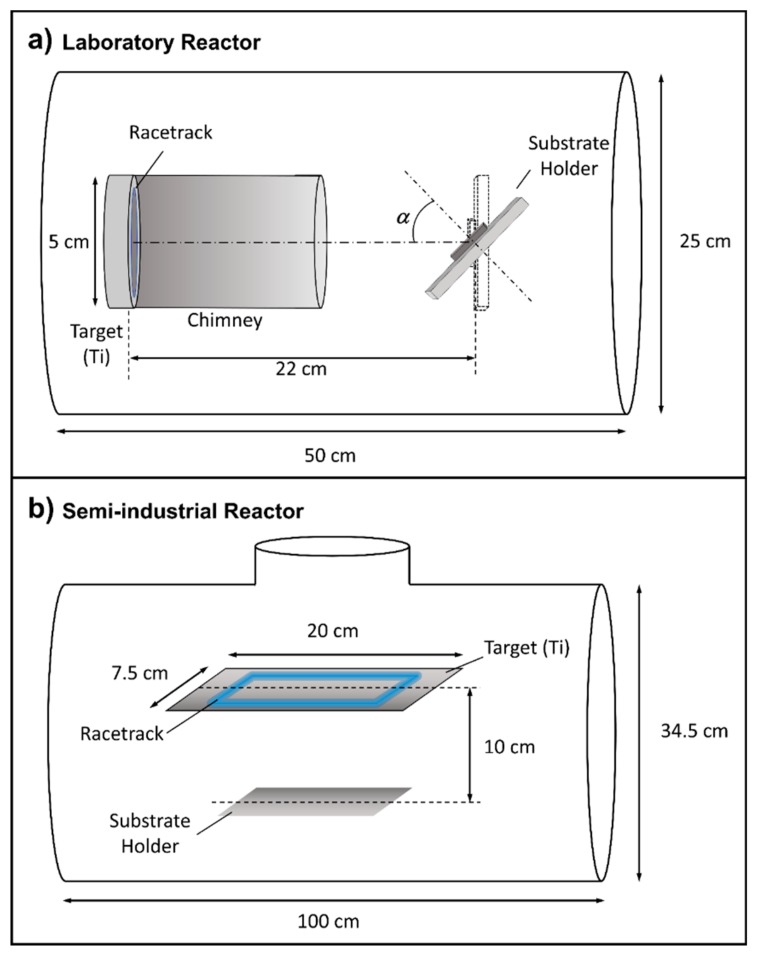
(**a**) Laboratory and (**b**) semi-industrial reactors employed to grow the Ti nanocolumns.

The Ti coatings were first grown in a MS laboratory setup described in detail in reference [30] that from now forth will be dubbed l-reactor (see Figure 1a). It has a magnetron head (AJA Inc., MA, USA) with a circular 5 cm diameter Ti target and a cylindrical 9 cm long metallic chimney that collimates the flux of sputtered material and traps many of the thermalized atoms. The base pressure in the reactor is in the order of 10^−7^ Pa and the distance between target and substrate is 22 cm. The parameters used to fabricate the columnar coatings in this reactor with Ar as sputter gas [30] are: Pressure = 0.15 Pa, power (DC discharge) = 300 W, and tilt angle of the substrate with respect to the target α = 80°. The semi-industrial scale reactor, which will be called i-reactor hereafter, operates at the company Nano4Energy (see Figure 1b). The target is rectangular and much larger (20 × 7.5 cm^2^) and, as a result of its balanced magnetic configuration, exhibits a racetrack with the shape of a rectangle (the long and short sides being 13.5 and 4.2 cm, respectively) with lines that are about 3 mm wide.

As a first step to scale up the growth conditions from the l-reactor to the i-reactor, we have employed as substrates fixation plates used in open trauma fractures that are known for their high postoperative infection rate (15% for patients with good health and more than 20% if they belong to risk groups). We coated two different fixation plates provided by Dr. Ricardo Larrainzar, Head of the Orthopedic Surgery and Traumatology Department at the “Infanta Leonor” University Hospital, Madrid. One of them was a new tubular plate from DePuy Synthes (made of stainless steel with length 5.2 cm, width 0.9 cm, and thickness 1 mm, with convex and concave sides), while the other was a pseudo-rectangular plate extracted from a patient and properly sterilized (with length 12 cm, width 1.3 cm, and thickness 4 mm). For depositions in the i-reactor, we used the following methodology: In the first stage, the plate was immersed in the plasma for cleaning purposes (pulsed DC voltage at 150 KHz, −500 V bias voltage, and a pressure of 1.2 Pa), after which the plate was left to cool down for 30 minutes. In the second stage, the Ti coating was deposited using the particular geometrical configuration presented in the Results and Discussion section. The deposition conditions were: Ar pressure = 0.4 Pa, power (DC discharge) = 325 W, and time = 25 min. Under these conditions, the deposition rate was ~12 nm/min and the thickness of the films about 300 nm. Finally, for the competitive studies between bacteria and osteoblasts, medical grade Ti-6Al-4V disks were also coated in the i-reactor and used for comparison with the large area sample results.

The microstructure of the coatings was studied with two different techniques: Scanning electron microscopy (SEM) with a Verios 460 field emission microscope (FEI Company, Hillsboro, OR, USA) using secondary electron detection, and atomic force microscopy (AFM) with a Dimension Icon microscope (Bruker Corporation, Billerica, MA, USA) that operates in a non-contact mode and type PPP-FM commercial probes,) (Nanosensors, Neuchâtel, Switzerland). 

To check the antibacterial properties of the coatings, the DePuy Synthes bone plate was introduced in a solution of *S. aureus* bacterial strain (10^8^ bacteria mL^−1^) (15981 laboratory strain, ATCC, Manassas, VA, USA) and incubated for 24 h in a 66% tryptic soy broth (TSB) + 0.2% glucose environment to promote biofilm formation (20 g L^−1^ of Difco Bacto TBS (Becton Dickinson, Sparks, MD)). After 24 h, the plate was washed three times with sterile phosphate-buffered saline (PBS), stained with 3 μL of SYTO-9/propidium iodide mixture, incubated for 15 min and washed with PBS. To determine the formation of the biofilm, we used calcofluor, a fluorescent dye that has been used to stain the biofilm extracellular matrix. In this case, 1 mL of calcofluor solution (5 mg mL^−1^) was used after the addition of the SYTO-9/propidium iodide mixture and was incubated for 15 min at room temperature. The formation of the biofilm was examined using a SP2 confocal laser scanning microscope (LEICA, Wetzlar, Germany). In this way, live and dead bacteria could be distinguished, with green and red, respectively, as well as the extracellular matrix of the biofilm with blue. Further details can be found in reference [10].

To further evaluate the antimicrobial activity of the nanostructured coatings, we carried out osteoblasts–bacteria competitive in vitro studies using the coated and uncoated regions of Ti-6Al-4V disks described above. For this purpose, co-cultures of MC3T3-E1 preosteoblast-like cells from mice (Sigma-Aldrich, San Luis, MO, USA) [31] and *S. aureus* [10] were co-cultured over uncoated surfaces and on surfaces coated with the Ti nanocolumns. Two different scenarios were simulated: i) Accidental infection (*S. aureus* concentrations of 10^2^ cfu/mL), and ii) osteomyelitis scenario (*S. aureus* concentrations of 10^6^ cfu/mL). In both cases, the *S. aureus* suspensions were mixed with 10^4^ cells/mL of MC3T3-E1 preosteoblast, suspended in Todd Hewitt broth (THB) and complete Dulbecco’s modified Eagle’s medium (DMEM) and simultaneously seeded on the samples. After 6 h of culture, confocal microscopy studies were done and lactate dehydrogenase (LDH) levels were measured as a parameter of osteoblast destruction. In this regard, for confocal microscope, the actin of preosteoblast cytoskeleton was stained with Atto565-conjugated phalloidin (red) and both cell nuclei and bacteria were stained with DAPI (blue). Moreover, LDH level was determined in the culture medium, which is directly related to the rupture of the plasmatic membrane, (cell death) which, when broken, releases all organelles and enzymes present in the cytoplasm. Measurements were performed by using a commercial kit (Spinreact, Girona, Spain) having an absorbance at 340 nm with a UV–Visible spectrophotometer. Two measurements of three independent experiments were carried out. All data are expressed as means ± standard deviations of a representative of three independent experiments carried out in triplicate. Statistical analysis was performed using the Statistical Package for the Social Sciences (SPSS) version 19 software (IBM, Armonk, NY, USA). Statistical comparisons were made by analysis of variance (ANOVA). Scheffé test was used for post hoc evaluations of differences among groups. In all of the statistical evaluations, *p* < 0.05 was considered as statistically significant. The most representative confocal images are shown in this study.

## 3. Results and Discussion

### 3.1. From Laboratory to Industrial Reactors

**Figure 2:**
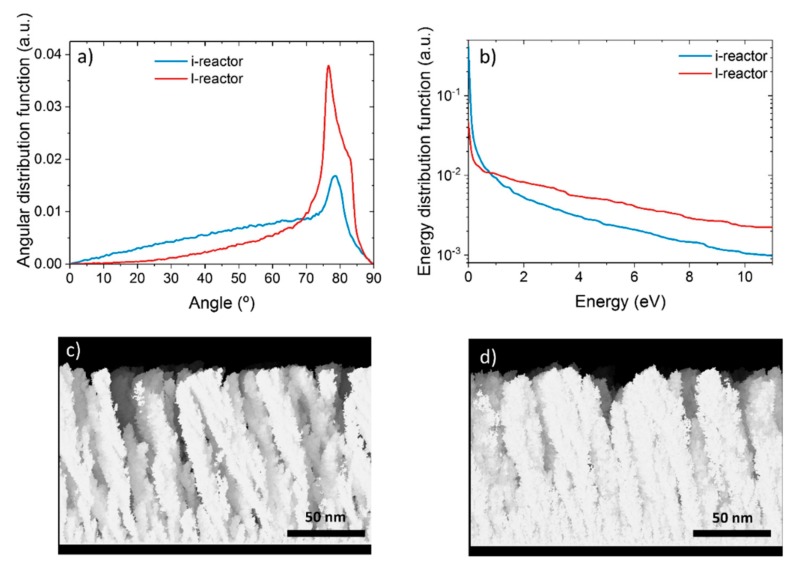
First row: (**a**) Polar angle distributions and (**b**) kinetic energy distributions of incident Ti atoms with respect to the surface normal in the l- and i-reactors (i.e., laboratory scale and semi-industrial scale, respectively). Second row: Solution of the model for the conditions in (**c**) the l-reactor and (**d**) the i-reactor.

In order to scale up the deposition procedure developed in the l-reactor we employed a well-known model to analyze the conditions required to grow the nanocolumnar films. In Figure 2a, we show the polar angle of incidence of sputtered Ti atoms on the tilted substrate in the l-reactor in our experimental conditions, as obtained by the SIMTRA code [32,33]. There, we can appreciate that the most probable angle of incidence over the substrate in this configuration is ~80°, which is above the calculated angular threshold of about ~70° required to promote the formation of the nanocolumns [30,34]. Moreover, as indicated in Figure 2a, there is a fraction of deposition species that arrives with lower angles of incidence, which corresponds to those atoms that have experienced collisions in the plasma and have altered their original steering [35]. The kinetic energy distribution function of these Ti atoms is shown in Figure 2b, and it is characterized by a long tail that extends up to energies above 10 eV, where the existence of numerous deposition species with kinetic energy above surface binding energy of Ti (~5 eV) is clear, i.e., with enough energy to induce kinetic energy-induced displacement processes of surface atoms upon deposition [30]. Using these distributions, we solved the model developed in reference [30] to account for the growth of Ti thin films by MS. The solution, presented in Figure 2c, shows a typical nanocolumnar array very similar to those experimentally obtained in reference [30], supporting that the necessary conditions for the growth of the Ti nanocolumns on a flat surface, as reported in reference [10], must also hold in the present case: i) The preferential angle of incidence of Ti sputtered species onto the surface must be centered at about ~80° with respect to the substrate normal, and ii) the kinetic energy distribution of deposition species must contain a significant fraction of atoms with energies above the binding energy of Ti surface atoms, i.e., ~5 eV.

**Figure 3:**
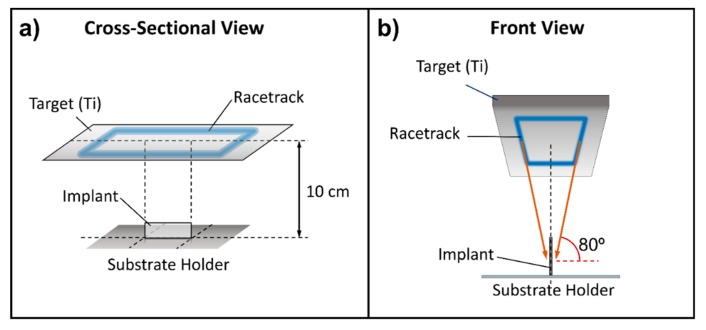
Proposed geometry ((**a**) cross-sectional and (**b**) front views) to coat the implants on two sides simultaneously with Ti nanocolumns.

Based on the results outlined above, we focused on reproducing both angular and kinetic energy distribution functions when operating the i-reactor on larger surfaces. In this way, and given the target and reactor geometry, we propose the geometrical arrangement shown in Figure 3. There, we placed the substrate perpendicular to the target, in such a way that atoms steaming from the racetrack may reach the substrate along an oblique angle of ~80°. Moreover, this particular configuration ensures that both sides of the substrate could be coated simultaneously. In Figure 2a, we show the calculated profile of the incident angle distribution of Ti species under this new configuration, where we can notice the similarities with that obtained in the l-reactor. This similarity extends to the kinetic energy distribution functions (see Figure 2b). In Figure 2d, we also show that the calculated nanostructure of the films in the i-reactor is formed by a nanocolumnar array, very similar to that obtained in the l-reactor (Figure 2c), suggesting the adequacy of the geometrical approach presented in Figure 3.

### 3.2. Coating the Tubular Plate from DePuy Synthes

**Figure 4:**
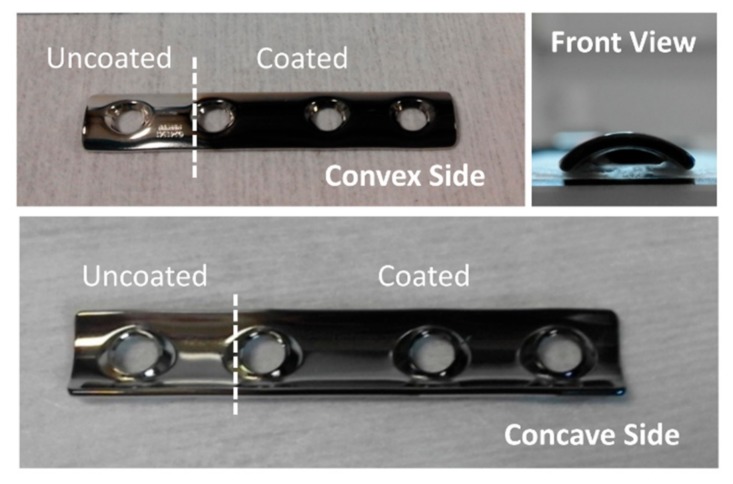
Different views of the DePuy Synthes plate coated in the i-reactor. A mask protecting about a quarter of the plate was used in order to have an uncoated zone to allow for comparison when performing in vitro analyses.

Following the geometrical configuration presented in Figure 3 and the conditions described in the Experimental Setup section, we coated the DePuy Synthes plate in the i-reactor. A mask protecting circa a quarter of the plate was employed to have an uncoated zone for the sake of comparison when performing in vitro analyses (see Figure 4). Scanning electron microscopy (SEM) and atomic force microscopy (AFM) were used to characterize the morphology of the coating, although the latter technique could only be applied on the convex side, as the tip holder of the microscope crashed with the lateral edges of the plate when approaching the concave surface. The uncoated zone presented a mirror-like brightness, indicative of small roughness. In agreement with this visual observation, both SEM and AFM images of this zone (not shown) indicate the absence of gaps or noticeable bumps on the surface, while the RMS roughness measured with the latter technique was 4 nm.

**Figure 5:**
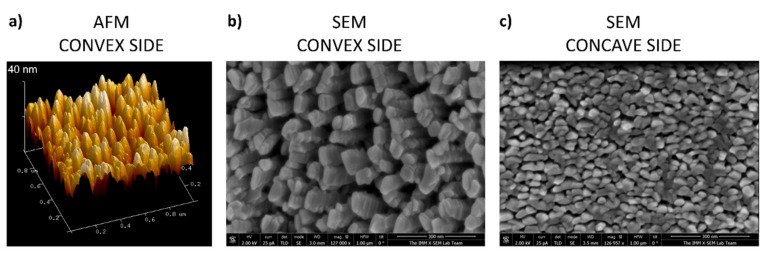
Microscopy images of the DePuy Synthes plate after deposition of Ti nanocolumns: (**a**) Atomic force microscopy (AFM) topographic map of the convex side of the plate; (**b**) SEM image of the nanocolumnar structures in the convex and (**c**) concave side of the plate.

Figure 5a shows an AFM topographic map taken on the coated zone (convex side of the plate). It is worth noting the good homogeneity of the coating and the existence of a microstructure that consists of regularly separated nanocolumns. Figure 5b,c shows SEM images of the coating that were obtained on the convex and concave sides of the plate. The former shows a well-distributed and homogeneous Ti nanocolumnar array, very similar to those arrays obtained under laboratory conditions in references [10,30]. However, on the concave side, even though the coating is also homogeneous and consists of nanocolumns, these are now smaller both in diameter and length and are well packed, resembling a film with a rather compact structure. This means that, due to the curvature of the concave side of the substrate, sputtered atoms arrive at the surface with an angle of incidence below 80° at some locations, resulting in structures similar to those found in the l-reactor for lower angles of incidence [30]. This difference could be minimized by placing the substrate closer to the target in Figure 3b, thus promoting the arrival of sputtered species along higher polar angles of incidence.

### 3.3. Coating of Pseudo-Rectangular Plate Extracted from a Patient

**Figure 6:**
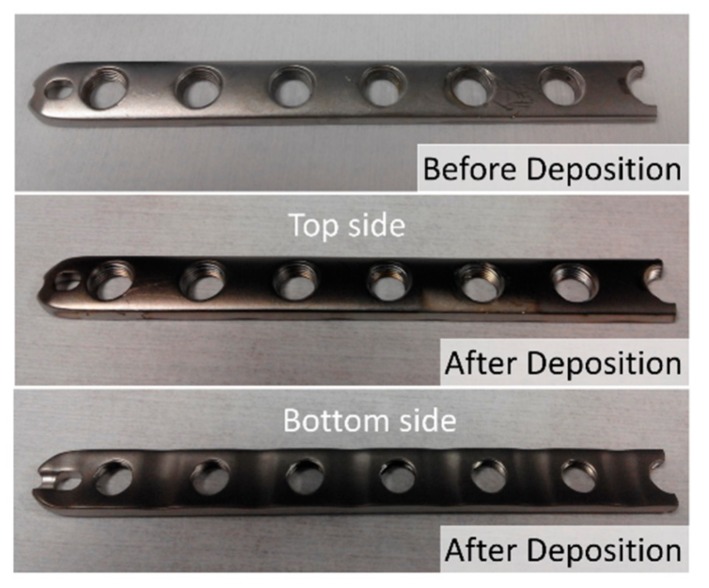
Photographs of the pseudo-rectangular plate extracted from a patient, before and after deposition of Ti nanocolumns.

**Figure 7:**
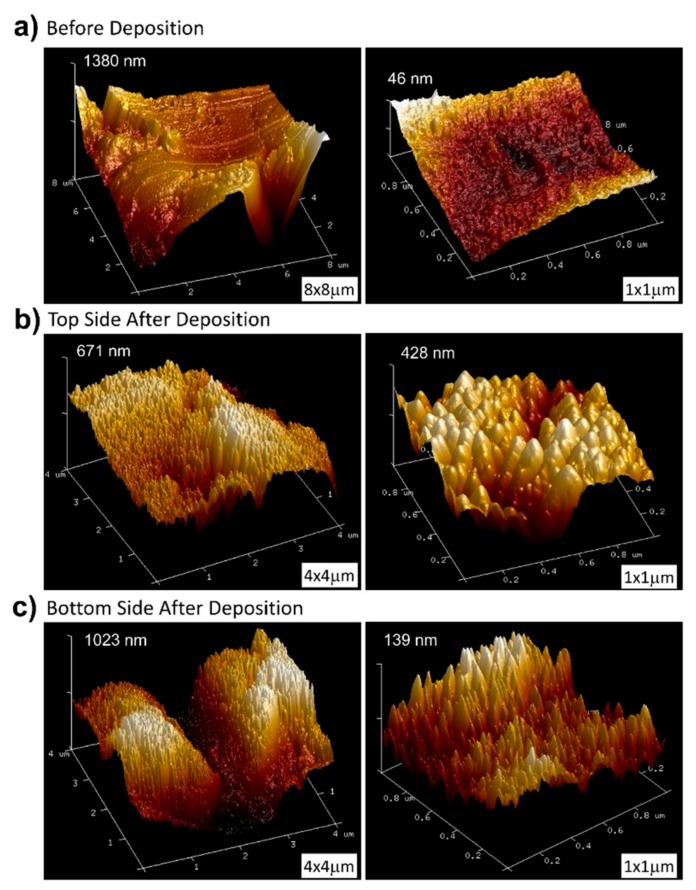
AFM images of the pseudo-rectangular plate extracted from a patient, obtained in areas with different size (left and right of the figure, respectively): (**a**) Before deposition; (**b**) top side after deposition; and (**c**) bottom side after deposition.

As an additional test of the geometrical arrangement presented in Figure 3, we analyzed the microstructure and morphology of a pseudo-rectangular plate extracted from a patient, as described in the Experimental Setup section. This plate was so large that it did not fit the entrance gate of the observation chamber of the SEM equipment and could only be analyzed by AFM. The initial gloss of the plate, which is rather matt instead of mirror-like, indicates that its roughness is high [36] (see Figure 6). To ascertain this, we performed a study of its morphology before the coating process. Figure 7a shows representative AFM images obtained in areas with different scale sizes (left and right of the figure, respectively) before deposition. On the higher magnification scale, the plate has a RMS roughness of 7 nm. However, in the image obtained in the same area but over a wider field of view (8 micron side), it can be seen that these flat areas are separated by deep cracks, with depths above one micron. This implies that, after the deposition process, most cracks will remain uncovered because their walls cast a shadowed region that avoids the arrival of most atoms inside, preventing the formation of nanocolumns. Consequently, the coatings will be inhomogeneous and there will be a large part of the implant surface (i.e., smooth areas of the initial surface) exhibiting well-formed nanocolumns, while a small percentage of it (deep cracks) will remain uncoated.

Following the sputtering process, the surface of the plate darkened considerably (c.f., middle and bottom panel in Figure 6), which indicates that a nanostructured coating has been successfully formed on both sides [37]. Figure 7b,c contains representative AFM images of the obtained coatings on both sides of the implant, upper and lower, respectively. They are composed of titanium nanocolumns, with a non-uniform distribution that depends on the morphology of the plate in each specific region: The columns grown on flat areas do have the same height, but those grown on the walls of the holes have lower height, as the initial surface was deeper. For example, Figure 7c shows an area with a very deep crack (depth about 1 micron) where it can be appreciated that the height of the columns is maximum at the top and gradually decreases when moving into the crack, until no columns are formed at the bottom. Overall, the columnar morphology of the coating is remarkably similar to that obtained on small substrates in the l-reactor in references [30] and [10].

### 3.4. Bacterial Adhesion and Biofilm Formation

**Figure 8:**
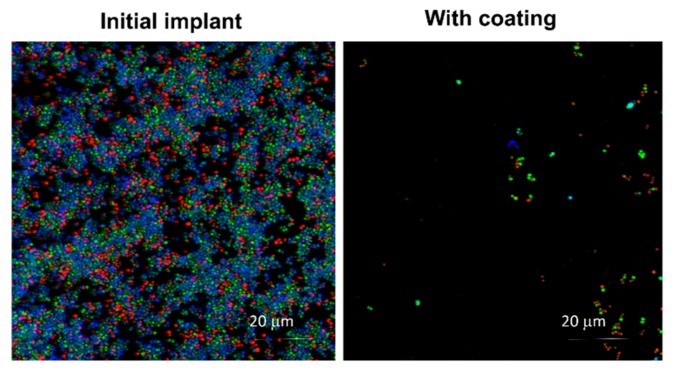
Antimicrobial activity in an osteoblasts–bacteria competitive in vitro scenario. Green corresponds to live bacteria, red to dead bacteria and blue corresponds to the extracellular matrix of the bacterial biofilm.

Once we had checked the nanocolumnar topography of the coatings produced in the i-reactor, we analyzed whether these maintain the same functionality as those produced in the l-reactor, i.e., if they are biocompatible and possess antibacterial capability. Following the bacterial growth procedure described in the Experimental Setup section, live and dead bacteria could be distinguished, with green and red, respectively, as well as the extracellular matrix of the biofilm with blue. Results appear in Figure 8, where we can clearly notice the bacterial proliferation on the uncoated region of the plate, which contains numerous living and dead bacteria, along with numerous blue staining, typical of extracellular matrix on the bacterial colonies. However, this blue stain does not appear in the coated zone in Figure 8, indicating the absence of bacterial biofilm in this case.

In order to further evaluate the antimicrobial activity of the nanostructured coatings, osteoblasts–bacteria competitive in vitro studies, already described in the Experimental Setup section, were also carried out in two different scenarios using the coated and uncoated regions of Ti-6Al-4V disks.

#### 3.4.1. Accidental Infection Scenario

**Figure 9:**
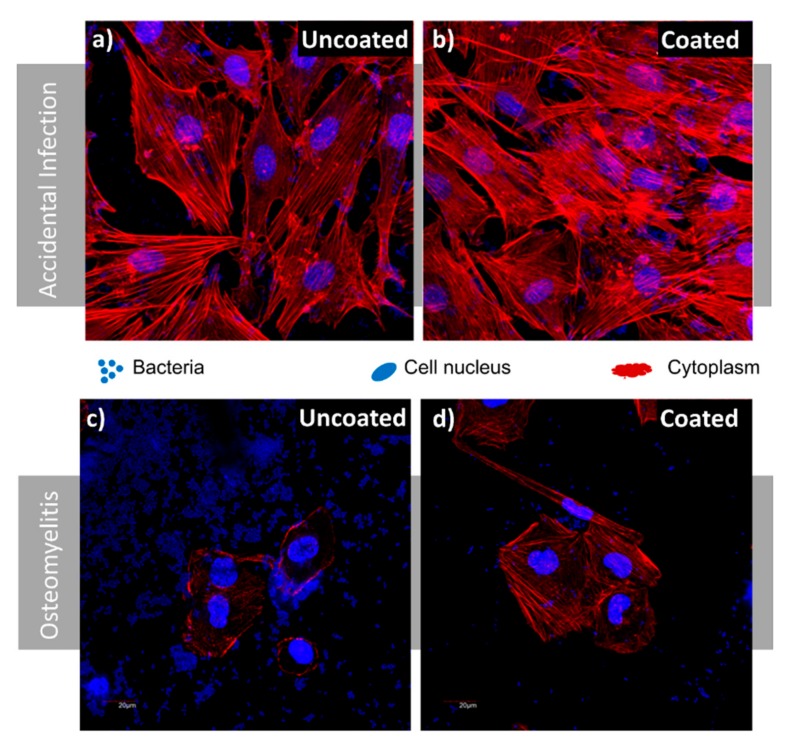
Competitive co-culture MC3T3-E1/*Staphylococcus aureus*: (**a**) 100:1 ratio (accidental infection scenario), uncoated region after 6 h; (**b**) 100:1 ratio (accidental infection scenario), coated region after 6 h; (**c**) ratio 1:100 (osteomyelitis scenario), uncoated region after 6 h; (**d**) ratio 1:100 (osteomyelitis scenario), coated region after 6 h.

**Figure 10:**
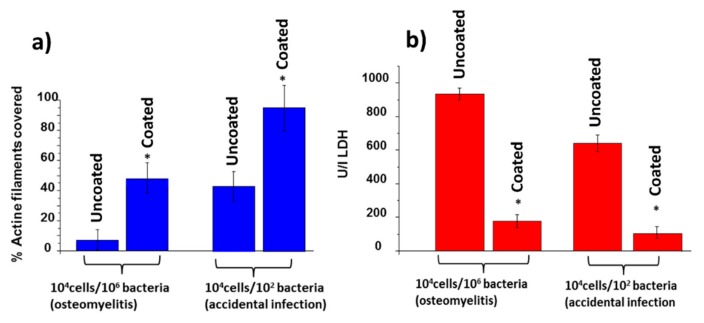
(**a**) Fraction of surface covered by preosteoblasts after 6 h under osteomyelitis (left) and accidental infection (right) scenarios; (**b**) lactate dehydrogenase (LDH) levels after 6 h under osteomyelitis (left) and accidental infection (right) scenarios.

In this first case scenario, the MC3T3-E1/*S. aureus* ratio seeded was 100:1. Good osteoblast adhesion was observed in the uncoated and coated surfaces (Figure 9a,b). However, several lacunae could be observed in the case of the uncoated surface (Figure 9a), were colonies of *S. aureus* were present. On the contrary, the nanocolumnar surface appears almost fully coated by a MC3T3-E1 preosteoblast-like cells monolayer that reaches about 90% coverage, as can be seen in Figure 10a (right).

LDH levels were measured as a parameter of cell destruction, illustrated in Figure 10b (right). There, it is evidenced that preosteoblast cell destruction is much higher on the uncoated surface than on the nanocolumnar coating under accidental infection scenarios.

#### 3.4.2. Osteomyelitis Scenario

In this case, the MC3T3-E1/*S. aureus* ratio seeded was 1:100. After 6 h of culture, Ti-6Al-4V was covered by a significant amount of bacteria that had colonized most of the implant surface (Figure 9c,d). The number of osteoblast cells was significantly reduced and the cells exhibited rounded morphology with a low spreading degree. On the contrary, the nanocolumnar surface showed a higher degree of osteoblast proliferation and spread, thus occupying a significant amount of surface (around 50% as observed in Figure 10a, left). The very low presence of *S. aureus* in this sample, compared with Ti-6Al-4V must be highlighted. The LDH measurements also evidenced much higher preosteoblast destruction in the case of Ti-6Al-4V (see Figure 10b, left).

As a final comment, it is important to underline that the existence of the cracks on the fixation plates reported above implies that the coating is not fully homogeneous and, therefore, based on the results presented in [10], its efficiency as an antibacterial coating can be affected. This issue could be minimized by making use of a rather standard industrial technique, by which the substrate rotates around a certain axis to enhance the film homogeneity. In this manuscript, we have not attempted this approach, as we aim at scaling up an already reported laboratory technique that operates on static substrates. Nevertheless, it is likely that the existence of cracks is minimized when the substrate rotates around an axis parallel to the target (parallel to the substrate holder line in Figure 3b), so sputtered species may arrive at the film following a constant polar angle of incidence, but different azimuthal angles.

## 4. Conclusions

We developed a methodology based on a new geometry to coat two-sided surfaces with areas up to ~15 cm^2^ with Ti nanocolumns by magnetron sputtering at oblique angles, and demonstrated its feasibility using a semi-industrial-scale reactor. This method was developed by calculating the necessary conditions for the growth of these structures in a laboratory-size reactor and reproducing them in a different geometry, suitable to coat larger areas in an industrial-scale reactor. These conditions were defined to control the incident angle distribution function of Ti atoms in the gaseous phase in such a way that they arrive at the surface along an oblique direction of about ~80–, and they possess a kinetic energy distribution function with a relevant proportion of deposition atoms with energies above the surface binding energy of Ti on the film surface.

After checking the homogeneity and features of the nanocolumnar structures deposited on different fixation plates on both sides, we analyzed the antibacterial functionality of the coating and demonstrated its equivalence to those produced in a laboratory reactor. In particular, two kinds of experiments were performed: Analysis of bacterial adhesion and biofilm formation, and osteoblasts–bacteria competitive in vitro scenarios, the latter also named “Race for the Surface” competition. In all these cases, we showed the opposite behavior of these surfaces toward osteoblast and bacterial proliferation and demonstrated that the proposed method represents a valid approach to coat large surfaces on both sides in industrial reactors, maintaining the same properties as laboratory-produced coatings on much smaller surfaces.
